# Reshaping the scissors: From directed evolution to AI-driven design for enhanced Cas performance

**DOI:** 10.1016/j.bidere.2026.100124

**Published:** 2026-09-30

**Authors:** Xinrui Cheng, Weimin Ruan, Zheng Jin, Langda Suo, Yuehui Ma, Lin Jiang, Wenxin Zheng, Ning Li, Wenping Hu

**Affiliations:** a State Key Laboratory of Animal Biotech Breeding, Institute of Animal Science, Chinese Academy of Agricultural Sciences, Beijing, 100193, PR China; b School of Life Science, Henan University, Kaifeng, 475000, PR China; c Institute of Animal Sciences, Tibet Academy of Agricultural and Animal Husbandry Sciences, Lhasa, 850009, PR China; d College of Animal Science and Technology, Xinjiang Agricultural University, Urumqi, 830052, PR China; e Beijing Capital Agribusiness Group, Beijing, 100193, PR China; f School of Animal Science, Shanxi Agricultural University, Jinzhong, 030801, PR China

**Keywords:** Cas protein optimization, High-throughput screening and directed evolution, Rational design, Protein engineering, Artificial intelligence

## Abstract

CRISPR/Cas gene editing holds great promise for applications in agricultural breeding and gene therapy; however, native Cas proteins face bottlenecks such as off-target effects, PAM limitations, and delivery challenges. This review explores a three-tiered, progressive, and synergistic analytical framework comprising directed evolution, rational structural design, and integrated protein engineering. Following the logic of “random exploration—precision modification—system integration,” it systematically clarifies the principles, advantages, disadvantages, and complementary interactions among these three optimization techniques. By comprehensively mapping the entire technical pathway for Cas protein improvement, this study fills a gap in systematic analysis within the field and provides a theoretical framework for tiered, synergistic modification to support the development of high-performance editors. The paper further analyzes a novel research and development paradigm in which AI and protein language models profoundly empower Cas modification. It summarizes intelligent toolchains such as structure prediction, virtual mutation, and de novo design of artificial Cas proteins, and highlights the unique advantages of AI in overcoming the performance trade-offs inherent in traditional protein engineering. At the same time, it identifies existing challenges, such as data scarcity and insufficient model interpretability, and looks ahead to how multimodal gene-editing large language models and automated closed-loop experimental systems will drive the iterative development of miniaturized, broad-spectrum, and highly precise editing tools, thereby facilitating the clinical and industrial implementation of gene-editing technologies.

## 1 Introduction

As a vital tool in modern biology, gene editing technology has had a profound impact on fields such as life sciences, medicine, and agriculture. The CRISPR/Cas system, an emerging gene editing technology, has garnered widespread attention due to its high efficiency, precision, and ease of use [1]. The origins of the CRISPR/Cas system can be traced back to the 1990s, when scientists discovered an adaptive immune system in bacteria and archaea capable of degrading the DNA of invading viruses or bacteriophages through RNA-guided mechanisms [2,3]. As research progressed, the CRISPR/Cas system was adapted into a highly efficient gene-editing tool, with its core components comprising Cas proteins and single-stranded guide RNA (sgRNA) [4].

The CRISPR/Cas system can be classified into Type I and Type II systems based on the number of Cas proteins, and is further subdivided into seven classes (Types I–VII) [5]. Class I systems (Types I, III, IV, and VII) consist of a complex assembled from multiple Cas protein subunits [6]. In contrast, Class II systems (Types II, V, and VI) rely solely on a single, larger Cas protein that contains multiple functional domains [2,7]. Due to their unique structural advantages, Class II systems are simpler to operate and exhibit higher editing efficiency. Currently, the CRISPR/Cas system primarily comprises three types: Cas9 (Type II), Cas12a (Type V), and Cas13 (Type VI), and a series of new editing tools have been developed based on these. Among them, CRISPR/Cas9 has become the most widely used system due to its simple and efficient editing capabilities [8]. CRISPR/Cas9 technology uses sgRNA to guide the Cas9 nuclease to the target DNA sequence, enabling precise gene knockout or knock-in Refs. [9,10]. In addition, a variety of molecular tools have been developed based on the CRISPR/Cas system. Intracellular functional tools are primarily divided into two major categories: genome editing tools, represented by base editors (BE) and prime editors (PE); and transcriptional regulation tools, represented by the CRISPR activation/inhibition system (CRISPRa/i) (Fig. 1). Beyond these, other types of tools have also emerged, including those for epigenetic modification, RNA editing, and in vitro assays. These technologies enable various forms of precise gene modification to meet different gene editing needs [11,12].

**Fig. 1:**
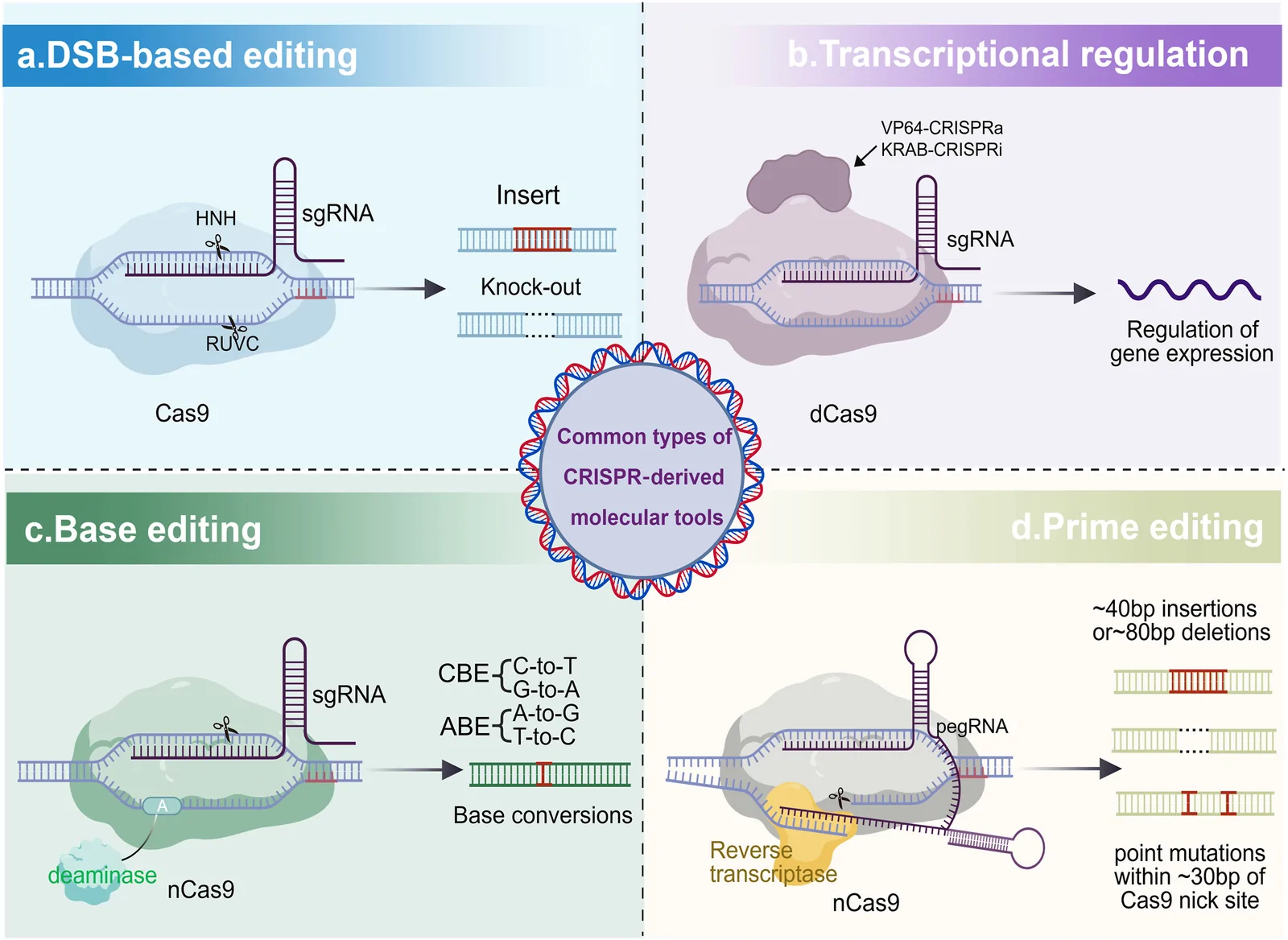
**Schematic illustration of four major classes of CRISPR-derived molecular editing tools. a.** DSB-mediated editing. Wild-type Cas9 guided by sgRNA generates DNA double-strand breaks (DSBs) via its HNH and RuvC nuclease domains. DSB repair through endogenous cellular pathways enables targeted gene knock-out or exogenous sequence insertion. **b.** Transcriptional modulators. Catalytically dead Cas9 (dCas9) loses nuclease activity; fusion with effector domains such as VP64 (CRISPRa, transcriptional activation) or KRAB (CRISPRi, transcriptional inhibition) achieves programmable modulation of endogenous gene expression without DNA cleavage. **c.** Base editors (BEs). A nickase Cas9 (nCas9) is fused to a deaminase domain. Cytosine base editor (CBE) mediates C•G-to-T•A conversion, while adenine base editor (ABE) catalyzes A•T-to-G•C base conversion, enabling precise single-base substitution without inducing DSBs. **d.** Prime editor (PE). The complex consists of nCas9 and reverse transcriptase, together with pegRNA that encodes both target recognition and desired edit information. Prime editing supports flexible genetic modifications, including small insertions (∼40 bp), small deletions (∼80 bp), and precise point mutations within approximately 30 bp flanking the Cas9 nicking site (Created with BioGDP.com [13]).

Although CRISPR/Cas9 has become the most widely used gene editing tool, its clinical translation remains constrained by four key bottlenecks: (1) off-target effects threaten treatment safety [14]; (2) PAM sites limit the scope of editing [15,16] ; (3) NHEJ-mediated repair produces unpredictable editing outcomes [17,18]; (4) the large molecular weight of Cas proteins leads to difficulties in in-vivo delivery [11,12] (Fig. 2, Table 1,Table 2). These bottlenecks are essentially closely associated with the intrinsic molecular properties of Cas proteins, which highlights the significance of Cas-protein optimization. Protein-engineering modifications of Cas proteins can alleviate the above-mentioned limitations in a targeted manner. For example, site-directed mutagenesis of domains such as REC and HNH can reduce off-target risks; modification of the PAM-binding pocket can relax target-recognition constraints; construction of nickases and diverse fusion proteins enables artificial regulation of editing outcomes; domain truncation and miniaturization can improve in-vivo delivery performance. Therefore, Cas-protein optimization serves as a general breakthrough to tackle these challenges in genome editing.

**Fig. 2:**
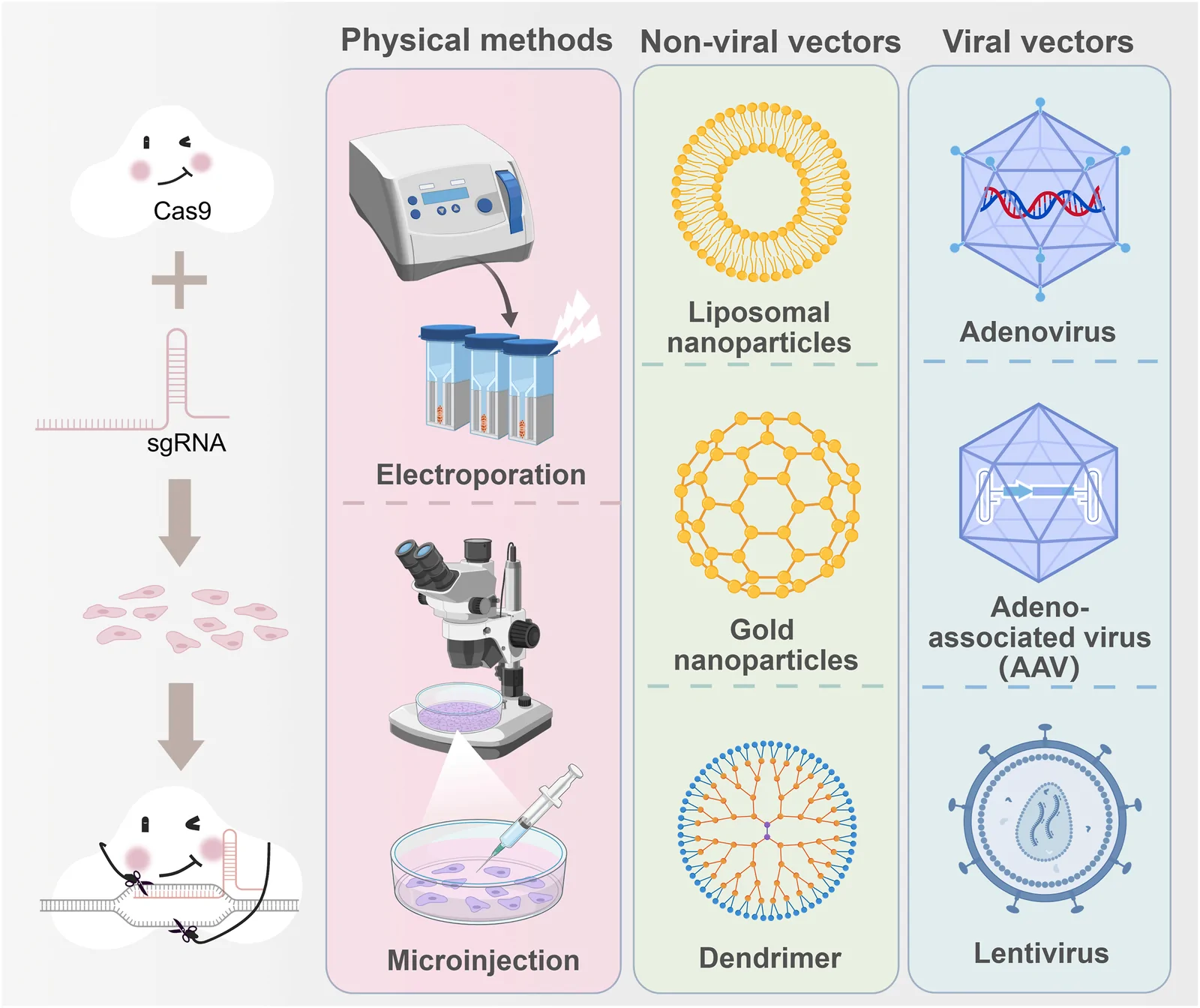
**Overview of major delivery strategies for CRISPR-Cas gene-editing components.** Schematic workflow of CRISPR-Cas9 system: Cas9 protein and sgRNA are delivered into target cells to exert genome-editing functions. Three primary delivery approaches are summarized in the figure. Physical methods include electroporation and microinjection, which directly introduce editing components into cells by physical force. Non-viral vectors mainly contain liposomal nanoparticles, gold nanoparticles and dendrimers, which serve as safe carriers to package and deliver CRISPR cargo. Viral vectors represented by adenovirus, adeno-associated virus (AAV) and lentivirus can efficiently deliver genetic encoding sequences of CRISPR-Cas9 into recipient cells (Created with BioGDP.com [13]).

**Table 1:** Comparison of CRISPR/Cas system delivery methods.

Delivery Options	Core Mechanism	Core Strengths	Major Weaknesses	Applicable Scenarios
Plasmid DNA (pDNA)	Intracellular transcription and translation produce Cas proteins	Stable, easy to build, low-cost, high-capacity	Slow onset of action, risk of genomic integration, and high off-target activity	Basic research on conventional cells,high-throughput screening
mRNA	The cytoplasm directly translates Cas proteins without a transcription step	Fast onset of action, no integration risk, and low off-target activity	Poor stability, prone to degradation, and dependent on carrier protection	CAR-T cell modification,rapid-onset local therapy
RNP Complex	Cas-sgRNA complexes pre-assembled in vitro enter cells directly and take effect	Rapid onset of action, low immunogenicity, zero integration, low off-target activity, and the highest safety profile	High costs, difficult encapsulation, and demanding delivery technology requirements	Primary cell editing, clinical cell therapy, high-precision gene editing

**Table 2:** Comparison of CRISPR/Cas system delivery vectors and technologies.

Delivery Categories	Specific Plan	Core Strengths	Major Shortcomings	Applications
Viral Vectors	AAV (Adeno-Associated Virus)	Safe, low immunogenicity, and highly tissue-specific	Very small package size	In vivo gene therapy, animal studies
	AdV Adenovirus	High transfection efficiency, high capacity, and no genomic integration	Highly immunogenic	In vivo targeted delivery, in vitro cell transfection
	LV Lentivirus	High efficiency, enables long-term, stable expression	Risk of genomic insertion mutations	Cells that are difficult to transfect,long-term expression for in vivo therapy
Non-viral Vectors	LNP Lipid Nanoparticles	Good biocompatibility, suitable for mRNA delivery	Overall stability is lacking	In vitro and in vivo local mRNA delivery
	Polymer Nanoparticles	Electrostatic encapsulation of nucleic acids results in high transfection efficiency	Some materials are highly cytotoxic	Routine in vitro cell delivery
	Inorganic Nanoparticles	High stability, easy to modify, suitable for RNP delivery	Biotoxicity, difficulty in metabolic clearance	Precision editing of RNPs in vitro
	DNA Nanocages	Excellent biocompatibility and stable RNP loading	Complex assembly, difficult to implement on a large scale	High-precision in vitro editing in the laboratory
Physical Delivery	Electroporation	High transfection efficiency, suitable for cells that are difficult to transfect	High pressure damages cells, low cell survival rate	High-throughput cell editing in vitro
	Microinjection	Extremely high precision, suitable for single-cell manipulation	Extremely low throughput, complex operation, and significant mechanical damage	Precision gene editing in embryos and single cells
Natural Biological Carriers	Exosomes/EVs	Low immunogenicity, natural targeting, and high biosafety	Low packaging efficiency, targeting needs improvement	Research and applications of safe gene delivery in living organisms

To improve the performance of Cas proteins, three major protein-engineering strategies have been established: (1) directed evolution, (2) rational/semi-rational design, and (3) multi-strategy integrated protein engineering. Directed evolution constructs mutant libraries and applies selective pressure to screen for Cas variants with improved performance [19]. Rational/semi-rational design performs site-directed mutagenesis or domain recombination at key functional sites based on protein three-dimensional structures and computational simulations [20]. Multi-strategy integrated protein engineering combines the strengths of the former two approaches. It relies on the closed-loop workflow of “screening-structural analysis-engineering modification” to realize the synergistic optimization of multiple protein traits. Each paradigm has its applicable scenarios and inherent drawbacks. Cross-technology integration has become a prevailing trend in this research field. Meanwhile, artificial intelligence technologies such as protein language models and deep learning act as cross-layer enabling tools throughout Cas-protein engineering. AI can intelligently design mutant libraries to boost the screening efficiency of directed evolution. Its predicted structural information can provide target references for rational design. Structural-biology analysis combined with wet-laboratory validation further elucidates the molecular mechanisms behind AI-predicted candidate variants [21]. Against this background, this review systematically traces the developmental trajectory of Cas-protein engineering from directed evolution and rational modification to AI-empowered optimization. It compares the principles, representative achievements, advantages and limitations of different strategies. It also discusses the prospects of multi-technology collaborative integration, so as to offer theoretical references for the research and development of next-generation high-performance genome-editing tools.

## 2 High-throughput screening and directed evolution:fundamental explorations for the optimization of gene-editing tools

Directed evolution is an optimization technique that mimics the laws of natural evolution and relies on human intervention to accelerate the iterative refinement of protein function. Its core features include random mutation, directed screening, multiple rounds of iteration, and the ability to operate without requiring knowledge of the complete structure. Even when the fine structure and mechanism of action of a protein are unclear, this approach can yield functionally improved variants through a trial-and-error cycle of mutation and screening [19]. High-throughput screening, as the core supporting technology for directed evolution, enables the rapid, quantitative screening of target candidate molecules from massive mutant libraries. The combination of these two techniques builds a fundamental trial-and-error platform for Cas protein optimization. This platform acts as a primary solution to overcome the inherent performance limitations of native Cas proteins at early research stages [22,23].

This strategy owns a standardized technical pipeline. Researchers construct large random mutant libraries via error-prone PCR, DNA recombination and transposon mutagenesis, which cover the PAM recognition and catalytic domains of Cas proteins.

Subsequently, fluorescent reporters [24], flow cytometry and high-throughput sequencing [25] quantify editing efficiency, PAM tolerance and off-target frequency of mutants. Continuous evolution tools including PACE [26,27] and CRISPR-MACE [28] are then employed to enrich advantageous mutations, achieving directional optimization of Cas performance.

The optimization and modification of Cas proteins require a synergistic approach combining high-throughput screening and directed evolution to efficiently identify high-quality variants, while the implementation of core steps—such as the construction of mutation libraries and the fine-tuning of candidate variants—ultimately relies on protein engineering techniques [29]. The core of directed evolution is a multi-round “mutation-screening” cycle. In this process, the construction of mutation libraries requires the design and implementation of protein engineering techniques—such as random mutation and site-directed mutation—to obtain a diverse library of variants that covers the target sequence space, thereby providing an ample pool of candidates for high-throughput screening (Fig. 3). Overall, this strategy leverages high-throughput screening to overcome the limitations of our understanding of regulatory mechanisms and is suitable for the initial modification of Cas proteins with complex structures and unknown functional mechanisms.

**Fig. 3:**
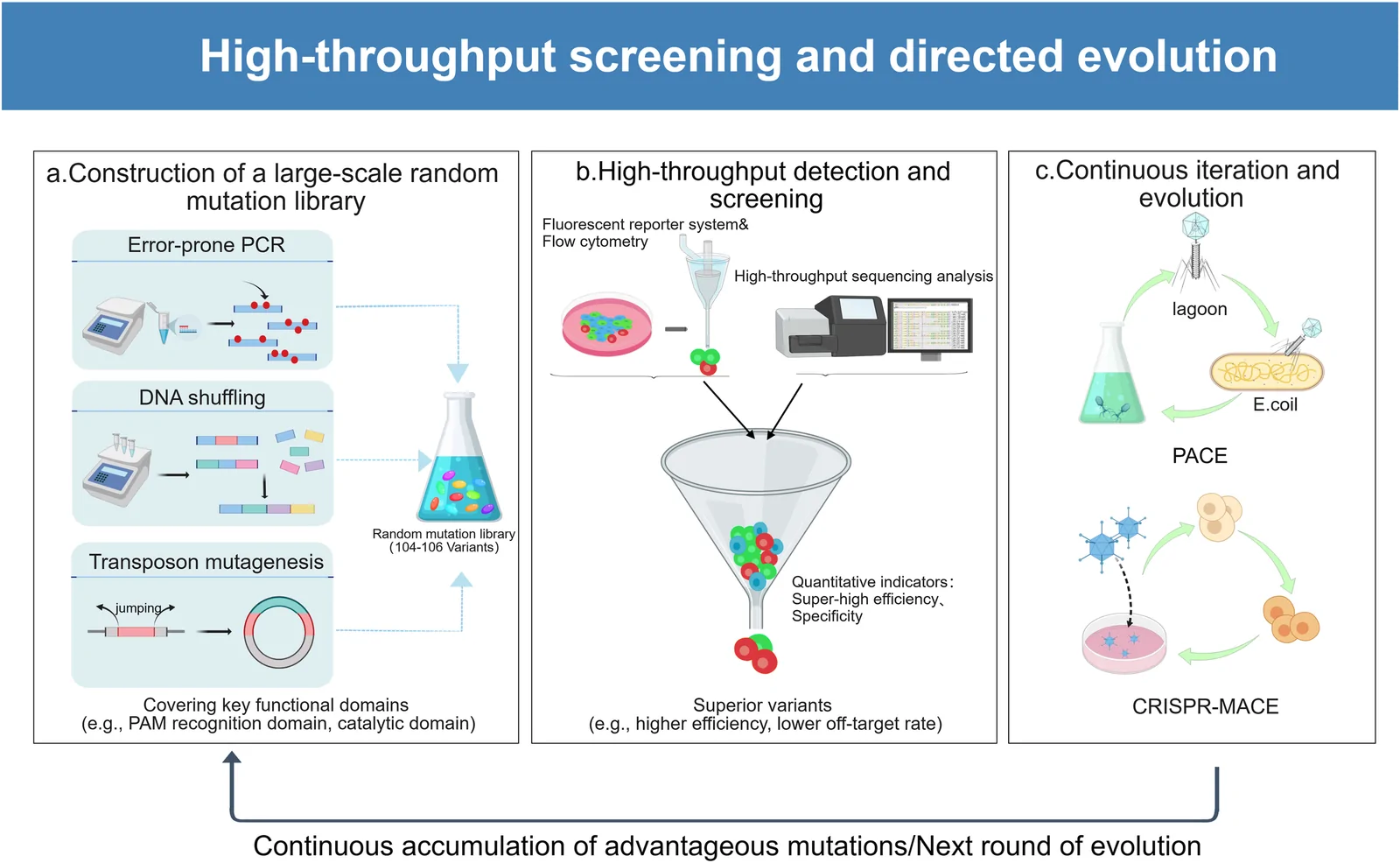
**Workflow of high-throughput screening and directed evolution for engineering CRISPR effector proteins. a.** Construction of large-scale random mutation libraries. Diversified mutant libraries containing 10^4-^10^6^ variants can be generated via error-prone PCR, DNA shuffling and transposon mutagenesis. Mutations are introduced to cover key functional domains of Cas proteins, such as the PAM recognition domain and catalytic domain. **b.** High-throughput detection and screening. Combined platforms including fluorescent reporter systems, flow cytometry and high-throughput sequencing are adopted for screening. Quantitative evaluation indicators mainly contain editing efficiency and targeting specificity, enabling enrichment of superior variants with improved performance (higher on-target activity and reduced off-target effects). **c.** Continuous iteration and evolution. Representative continuous evolution platforms include PACE and CRISPR-MACE, which realize autonomous iterative evolution without repeated manual library construction and screening. Beneficial mutations obtained from screening can be accumulated continuously to initiate the next round of evolutionary cycle(Created with BioGDP.com [13]).

### 2.1 A breakthrough expansion of PAM compatibility

The high specificity and narrow targeting range of the PAM sequences recognized by natural Cas proteins are the key bottlenecks limiting their application in genome editing. Traditional site-directed mutagenesis relies on structural knowledge and has limited room for improvement, whereas directed evolution—through unbiased random mutations and multiple rounds of screening—can effectively overcome the limitations of PAM recognition. Directed evolution studies of the classic SpCas9 have identified broad-spectrum PAM-recognition variants such as xCas9-3.7 [30], SpG, and Spry [31], which combine a wide targeting range with low off-target activity. For small Cas proteins—which have low molecular weight and significant delivery advantages but are structurally compact and difficult to rationally engineer—directed evolution can circumvent the challenges of structural analysis and directly screen for active variants that bind to a wide range of PAM sequences [32]. Some of these variants exhibit superior editing efficiency and specificity compared to their natural homologs, greatly expanding the application scenarios for small Cas proteins.

### 2.2 Overcoming the activity-specificity trade-off

The trade-off between editing activity and target specificity is a core, common challenge in Cas protein optimization; traditional rational design for a single target struggles to balance these two properties. In contrast, directed evolution enables joint screening across multiple criteria, allowing for the simultaneous optimization of multiple constraining traits. Mark A. Behlke's team utilized a dual-screening system (positive and negative) in *E. coli*, combined with the construction of a random mutation library, to iteratively evolve a HiFiCas9 variant. This variant integrates multiple synergistic beneficial mutations, significantly reducing off-target risks while maintaining high target editing activity [33]. In recent years, directed evolution coupled with high-throughput screening has enabled the generation of multiple high-performance, high-fidelity Cas9 variants, demonstrating the strengths of this strategy in balancing editing activity and specificity. Via iterative rounds of random mutagenesis and functional screening, evoCas9 was engineered with four key beneficial mutation sets to substantially lower genome-wide off-target rates while preserving high on-target editing efficiency [34]; the iteratively improved Sniper2L variant further addresses the issue of diminished activity in most high-fidelity Cas9 variants; it eliminates single-base mismatch off-target events while maintaining wild-type editing efficiency and is compatible with various clinical delivery systems [35]. Furthermore, the novel SpCas9-Mut5 variant, obtained through high-throughput functional screening, combines ultra-high specificity with lossless editing activity and can significantly suppress genomic structural abnormalities [36]; both the AI-assisted OpenCRISPR-1 and the screening-obtained FrCas9 have overcome the performance bottlenecks of traditional rational design via multi-site synergistic optimization, achieving simultaneous improvement of high activity and high fidelity [37,38]. Compared to the limitations of single-point modifications in rational design, the aforementioned series of studies collectively demonstrate that directed evolution can enrich optimal mutation combinations through natural selection of massive numbers of mutants, uncover multi-site synergistic effects that are difficult to predict through artificial design, and effectively resolve the core trade-off challenges in Cas protein optimization.

High-throughput screening and directed evolution provide a fundamental approach for achieving breakthroughs in Cas protein performance, but they have significant inherent limitations: first, the experiments are costly and time-consuming; constructing large-scale mutation libraries and conducting multiple rounds of screening require substantial research resources, with iteration cycles ranging from months to years; second, the improvement process is highly random, making it difficult to precisely control mutation sites and impossible to efficiently optimize multiple constrained traits simultaneously; third, there is a lack of mechanistic understanding; while performance-enhanced variants can be obtained, the functional regulatory mechanisms of the mutation sites cannot be elucidated, making it difficult to guide subsequent precise and systematic improvements.

## 3 Structure-based rational design: a precision breakthrough in gene-editing tool optimization

Structure-based rational protein design is a precision protein engineering method grounded in an in-depth understanding of protein structure and functional mechanisms. It differs fundamentally from the random trial-and-error approach of directed evolution, marking a transformative leap in Cas protein optimization from “blind screening” to “controlled design.” The core implementation logic of this strategy is as follows: using the high-resolution 3D structure of the Cas protein, gRNA, and target DNA complex as the fundamental premise, structural biology analyses are employed to identify key functional regions such as the catalytic center, PAM recognition domain, and DNA-binding interface, thereby pinpointing the amino acid residues that regulate editing activity, target specificity, and PAM compatibility; bioinformatics tools, including molecular dynamics simulations and energy calculations, are then used to precisely predict the functional effects of point mutations; finally, through targeted mutation engineering and functional validation, precise site-specific optimization of the Cas protein is achieved [20]. Compared to directed evolution—which does not require an understanding of the underlying mechanism and relies on extensive trial and error—rational design represents a major innovation in that it is guided by structural mechanisms and centered on precise site-specific modification, thereby fundamentally avoiding the blindness and randomness of random screening. The core technical characteristics of these two approaches are distinctly complementary.

From a technical workflow perspective, rational design follows a three-stage closed-loop pipeline: structural analysis, in silico screening, and precise experimental validation. This workflow is more concise, target-oriented and cost-effective for research and development than the trial-and-error pipeline of directed evolution, which relies on random library generation and large-scale phenotypic screening. First, structural analysis lays the foundational basis. Researchers acquire high-resolution structures of Cas–nucleic acid complexes via cryo-electron microscopy [39], X-ray crystallography [40] or nuclear magnetic resonance spectroscopy [41]. These structural datasets accurately map functional residues and molecular interaction hotspots, offering explicit sites for targeted mutagenesis. Unlike directed evolution, this remodeling strategy does not require prior structural information of target proteins. Second, computer-aided virtual screening acts as a central module. Researchers employ modeling platforms including PyMOL and Rosetta to simulate mutational impacts in silico. These simulations quantify how single substitutions, combinatorial mutations or structural rearrangements alter protein stability, substrate binding affinity and genome editing activity. Such prediction tools prioritize favorable mutant combinations, greatly reducing the scale of wet-lab tests and eliminating the heavy resource burden of large random libraries required for directed evolution [42]. Third, heterologous expression and functional characterization serve as the final experimental checkpoint. Researchers generate and express designed mutant proteins. A panel of multi-dimensional functional assays then evaluates their editing activity, target specificity and structural stability. This step completes the full closed-loop workflow of computational design and experimental verification (Fig. 4) [43].

**Fig. 4:**
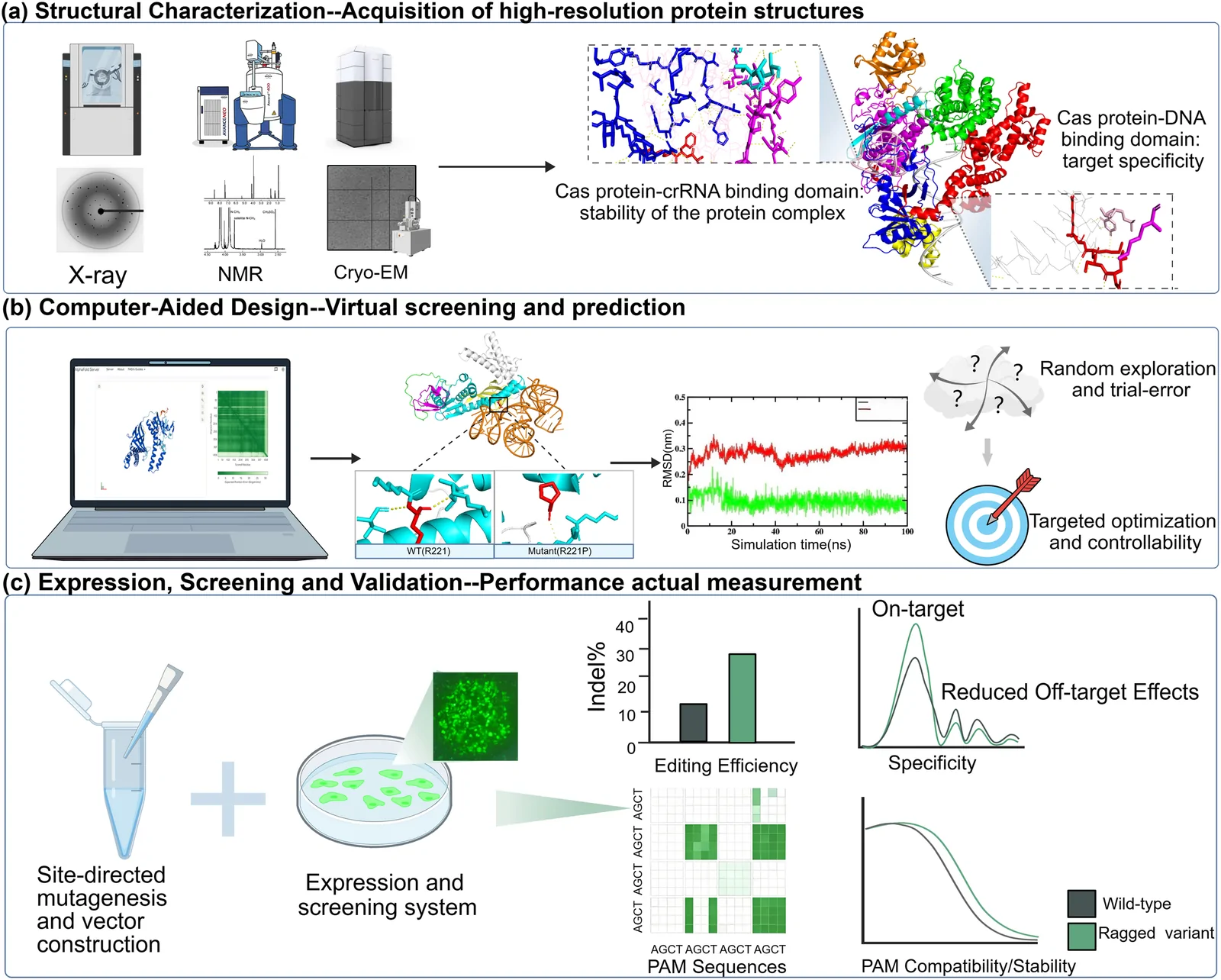
**Schematic workflow of structure-guided rational engineering for CRISPR Cas effector proteins. (a)** Structural characterization for resolving high-resolution protein structures. X-ray crystallography, nuclear magnetic resonance (NMR) and cryogenic electron microscopy (cryo-EM) are major techniques to obtain atomic-level structures of Cas protein complexes. Structural information reveals key regions including the Cas protein–crRNA binding domain responsible for complex stability, and the Cas protein–DNA binding domain that governs target recognition specificity. **(b)**Computer-aided protein design for virtual screening and prediction. Based on resolved three-dimensional structures, molecular modeling and molecular dynamics simulations are implemented to analyze intermolecular interactions. This strategy shifts engineering from random trial-and-error exploration toward targeted, controllable optimization of protein properties. **(c)** Experimental expression, screening and functional validation for quantitative performance evaluation. Site-directed mutagenesis and vector construction generate candidate variants, which are then expressed and tested in cell-based screening systems. Multiple indicators are measured to characterize engineered variants, including editing efficiency (Indel%), targeting specificity (suppressed off-target activity), PAM compatibility and protein stability, for comparison against wild-type Cas proteins(Created with BioGDP.com [13]).

In the Cas protein optimization framework, rational design offers irreplaceable technical value and synergistic advantages. Unlike directed evolution, which is limited to improving variants through phenotypic screening and struggles to elucidate mutation mechanisms, rational design can precisely analyze the functional regulatory mechanisms of mutations. It can not only independently carry out refined modifications of Cas proteins—such as enhancing specificity, regulating activity, reshaping function, miniaturization and streamlining, and species adaptation—but also empower directed evolution systems in a reverse manner: by structurally guiding the targeted construction of mutation libraries, it reduces screening costs and enhances iterative efficiency. At the same time, it enables secondary, precise optimization of high-quality variants obtained through directed evolution screening, further balancing the trade-off between activity and specificity. This represents the core technological framework for advancing Cas proteins from breakthroughs in basic performance toward precise, functional, and industrial-scale improvements.

### 3.1 Enhancing specificity: Structural mechanism analysis guides off-target risk management

Off-target effects are a key risk limiting the clinical translation of Cas proteins; directed evolution can only identify variants with low off-target activity through screening and struggles to address the issue at the mechanistic level. In contrast, both HypaCas9 and SuperFi-Cas9 are engineered through structure-guided rational design. HypaCas9 is based on the structural mechanism by which the REC3 domain of SpCas9 mediates sgRNA recognition and target DNA binding. It employs combinatorial mutations in amino acid residues of the REC3 domain (N692A, M694A, Q695A, H698A) to enhance targeting specificity [44]. SuperFi-Cas9, developed by the University of Texas team, relies on a rational design approach [45]. First, through structural biology, the team elucidated the molecular mechanisms underlying Cas9 off-target effects, identifying non-specific interactions at the DNA-binding interface and abnormal structural flexibility as the core causes of off-target effects; By making targeted fine-tuning of amino acid residues in the DNA-binding and catalytic domains, the team ultimately obtained high-quality variants with significantly reduced off-target probabilities and no loss of editing efficiency. This achieved a closed-loop optimization process of “mechanism analysis—precision modification—performance enhancement,” which, compared to random screening approaches, substantially reduced R&D costs and improved the precision of modifications.

### 3.2 Enhanced efficiency: Precise regulation of activity achieved through modification of key functional domains

The Doudna team's modifications to the Cas9 WED domain fully demonstrate the advantages of precise regulation through rational design. By resolving the dynamic structure of the Cas9-gRNA-DNA complex via cryo-electron microscopy, the team clarified the central regulatory role of the WED domain's DNA unwinding efficiency in the overall editing process and identified key functional amino acid sites. Through site-directed mutagenesis to optimize domain interaction patterns, they significantly enhanced DNA unwinding rates and overall editing activity; moreover, this modification strategy can be generalized to various Cas9 homologs, enabling family-wide activity improvement [46]. HyperDriveCas9 also employs structure-guided rational design; researchers modified relevant domains based on the structural characteristics of the Cas9 protein and the principles of editing, yielding variants with superior performance [47]. Furthermore, through rational approaches such as structural dynamic network analysis and surface charge modification, the activity, stability, and delivery compatibility of Cas proteins can be simultaneously regulated, achieving multidimensional precision optimization [48,49].

### 3.3 Functional conversion: Domain-targeted mutations for expanding tool functionality

Rational design can achieve Cas functional reengineering by targeting the functional domains of proteins, primarily through two independent modification strategies, thereby overcoming the inherent functional limitations of native Cas proteins. The first modification strategy involves site-specific inactivation of catalytic domains: classic studies have used site-specific mutations to inactivate the RuvC and HNH endonuclease domains of Cas9, yielding dCas9, which loses its ability to cleave double-stranded DNA but retains only the DNA-binding scaffold, thereby achieving a functional transition from a DNA-cleaving tool to a targeting scaffold protein. The second modification strategy involves the fusion and assembly of functional modules: by grafting exogenous functional protein domains onto the full-length nuclease nCas9 or the non-enzymatic dCas9 scaffold, entirely new biochemical activities are directly conferred. Subsequently, CRISPRa/i transcriptional regulatory systems were constructed through the modular fusion of dCas9 with the transcript activator VP64 and the repressor KRAB; various single-base editors were developed by fusing deaminases with nCas9, and precision micro-mutation modifications using prime editors in combination with reverse transcriptases were also achieved through this fusion strategy [50]. These two modification approaches complement each other, fully highlighting the core advantages of rational design: precise site-specific and directed modification, as well as the absence of redundant modifications.

### 3.4 Miniaturized design: Development of a compact editor suitable for AAV delivery

Miniaturization for use with clinical delivery vectors such as AAV is a key requirement for the industrialization of Cas proteins, a process that relies heavily on rational structural design. By analyzing structurally redundant regions of Cas proteins, researchers have precisely deleted and truncated non-essential domains while preserving core functional domains, leading to the development of compact editors such as MISER Cas9 [51] and mini-SaCas9 [52]. These miniaturized proteins have significantly reduced molecular weights, enabling efficient delivery via AAV vectors while retaining full editing activity, thereby addressing the industry-wide challenge of low delivery efficiency posed by naturally large Cas proteins. In addition to truncating and modifying classic large Cas homologs, the native Cas12f subfamily of nucleases represents another excellent platform for miniaturization. Optimization can be achieved through pure site-directed rational mutagenesis or semi-rational strategies combining cryo-EM structural analysis with iterative directed evolution. Native Cas12f contains only 400–700 amino acids, far fewer than SpCas9 (approximately 1368 aa) and LbCas12a (approximately 1300 aa). The complete expression cassette, comprising the protein and guide RNA, can be fully loaded into a single AAV vector, significantly simplifying the in vivo gene therapy workflow. The core objectives of Cas12f engineering focus on enhancing cleavage activity, broadening the PAM recognition range, and improving protein thermodynamic stability. Currently, several optimized variants with fully validated functionality are available: the AsCas12f variants yham and hkra were derived from deep mutation screening combined with protein structure-based selection; by modifying the dimerization interface and strengthening nucleic acid-binding interactions, they achieved a tenfold increase in activity without increasing the risk of off-target effects [53]; a team from Shanghai Jiao Tong University employed semi-rational iterative engineering to develop AsCas12f1-M5, which relaxes PAM recognition constraints and increases genomic coverage by 3–4-fold [54]; the University of Texas team engineered Al3Cas12f through structural remodeling to create Al3Cas12f RKK, which significantly improved cleavage efficiency at hard-to-target genomic sites while maintaining robust catalytic activity even at low viral doses [55]. The full suite of rationally engineered Cas12f variants described above has achieved breakthrough improvements in editing activity, PAM compatibility, protein stability, and in vivo adaptability with AAV, providing high-quality miniaturized tools for precision gene therapy. In addition, ultra-miniature transposon-derived TnpB/IscB nucleases, measuring only 350–410 amino acids, have been identified and engineered; these can serve as complementary platforms to further push the limits of viral vector loading capacity. In summary, under the framework of rational design, two technical approaches for editor miniaturization coexist in parallel: one involves removing redundant domains from large Cas proteins, and the other involves rational or semi-rational engineering of naturally small Cas12f and TnpB/IscB proteins. Both types of compact tools are suitable for in vivo delivery via a single AAV and represent indispensable branches of research in rational protein engineering.

### 3.5 Universal engineering and species-specific optimization of Cas proteins

Rational design can overcome the limitations of single-protein modification and enable large-scale, systematic improvement of the Cas protein family. Through multidimensional rational modifications—including codon optimization, functional module fusion, and signal peptide modification—a Chinese research team optimized the cellular expression efficiency and editing activity of Cas12i.3, achieving the capability for simultaneous multi-target editing using a single plasmid, thereby making it suitable for large-scale genome editing research [56]. The MIDAS modification method developed by the Chinese Academy of Sciences has achieved cross-family, universal improvements, enabling the efficient optimization of various CRISPR nucleases—including Cas12i, Cas12b, and CasX—and establishing a universal Cas protein modification system [57]. At the same time, addressing the differences in Cas protein activity across species, researchers used site-directed mutations to optimize the proteins’ adaptability to host nuclear transport and the chromatin environment, developing species-specific variants such as LbCas12a-RRV [58] and ttLbCas12a Ultra [59], thereby resolving the issue of uneven editing efficiency across different systems.

In short, structure-guided rational design enables multi-faceted optimization of Cas proteins via four distinct engineering strategies. First, site-directed mutagenesis targeting key functional residues markedly elevates Cas editing performance, based on structural dissection and residue functional annotation. Second, domain fusion links Cas scaffolds to effector moieties including deaminases, reverse transcriptases and transcriptional modulators. This strategy has yielded versatile editing platforms such as base editors, prime editors and gene regulatory tools. Third, targeted truncation removes structurally dispensable segments, generating compact mini-Cas variants compatible with AAV-mediated in vivo delivery. Fourth, codon recoding and signal peptide fusion boost heterologous expression and genomic targeting in mammalian, plant and microbial hosts, which greatly broadens the practical utility and applicable scenarios of CRISPR editing systems.

It is worth noting that structure-based rational design still has significant limitations. The core limitation of rational design lies in its heavy reliance on structural knowledge: high-resolution structures of protein-substrate complexes are required to carry out modifications, and the structures of most novel Cas homologs have not yet been determined, which severely limits their scope of application. At the same time, it is difficult to accurately predict the synergistic effects of multi-site mutations, making the synergistic optimization of multiple targets—such as a broad PAM, high activity, high specificity, and small size—extremely challenging; furthermore, structural determination and molecular simulations require specialized equipment and involve high technical barriers, limiting their widespread adoption.

## 4 Comprehensive protein engineering strategies: Systematic integration of optimized gene-editing tools

High-throughput directed evolution and rational structural design are significantly complementary technologies: the former excels at broadly mining functional variants and overcoming performance bottlenecks, but suffers from unclear mechanisms and a high degree of optimization uncertainty; the latter excels at precise, site-specific modifications, with clear mechanisms and strong targeting capabilities, but is limited by barriers to structural understanding and an inability to optimize multiple traits in concert. As a third-generation optimization framework, integrated protein engineering strategies focus on combining the broad-spectrum screening advantages of random selection with the precision of rational design. Through a closed-loop process—“initial screening to expand the scope, mid-stage structural analysis to identify targets, and late-stage engineering for integration”—these strategies overcome the technical shortcomings of individual approaches and achieve multidimensional, synergistic optimization of Cas protein activity, specificity, size, deliverability, and multifunctionality. This approach represents the mainstream strategy for developing high-performance Cas editing tools today.

The core practical value of this integrated strategy lies in resolving the inherent contradictions of single-strategy approaches and achieving synergistic performance improvements. The process by which the Huida Genomics team developed hfCas12Max is highly representative: first, relying on high-throughput metagenomic screening, they identified a naturally highly active xCas12i prototype protein, establishing a foundation of inherent activity; they then combined bioinformatics analysis with rational structural design to precisely modify key functional sites and optimize the protein's targeting specificity, ultimately yielding a Cas12Max variant that combines high editing efficiency with an ultra-low off-target risk [60], thereby circumventing the mechanistic shortcomings of pure screening and the activity limitations of prototype proteins derived solely from rational design.

The Doudna team's optimization of Flex-Cas12a further validated the advantages of the integrated strategy: first, through directed evolution, they identified the Lbm6 variant with significantly enhanced PAM compatibility, overcoming the targeting limitations of native Cas12a; then, relying on rational structural design and incorporating activity-enhancing site-specific mutations, they increased the variant's editing efficiency from 34% to 80%, achieving dual breakthroughs in both PAM range and editing activity [61]. Compared to a single-optimization approach, the integrated strategy precisely balances the trade-offs among multiple performance metrics, ensuring editing safety while expanding the editing scope, and is thus better suited to the stringent requirements of clinical translation.

Integrated protein engineering, which combines the advantages of directed evolution and rational design to achieve synergistic optimization of multiple traits in Cas proteins through a closed-loop “screening–structure determination–modification” process, is the mainstream approach for developing high-performance editing tools; however, it still has significant limitations. This strategy is constrained by shortcomings in upstream technologies: the construction of directed evolution libraries and multi-round screening are time-consuming and labor-intensive, while rational design relies on the structural elucidation of Cas protein–nucleic acid complexes. However, structural information is lacking for many novel homologous proteins, limiting target discovery. At the same time, manual methods cannot exhaustively explore the vast number of mutation combinations; in optimization scenarios involving multiple coupled objectives—such as broad-spectrum PAM recognition, high activity, low off-target effects, and miniaturization—it is difficult to fully exploit the synergistic effects of multi-site mutations. Furthermore, traditional experimental systems cannot create entirely new Cas scaffolds independent of natural templates, and tool innovation is constrained by natural protein sequence resources. These bottlenecks in traditional engineering approaches have propelled artificial intelligence and protein language models to become a new paradigm for Cas protein modification.

## 5 Engineering enhancements to the Cas editor based on artificial intelligence and protein language models

With the advancement of artificial intelligence technologies (such as protein language models and structure prediction models), the optimization and modification of gene editors will become deeply integrated with AI: AI-assisted high-throughput screening enables the intelligent design of mutant libraries, reducing the scale of screening [[62], [63], [64]]; AI-driven structure prediction can rapidly determine the three-dimensional structures of unknown proteins, lowering the threshold for rational design [65]; AI-mediated protein engineering enables global prediction for multi-objective optimization [[66], [67], [68]]. The advancement of AI has also driven the deeper application of synthetic biology in the field of gene editing, such as the “de novo design and synthesis” of gene editors. Rather than relying on existing editors discovered in nature, this approach utilizes trained large-scale AI protein models to directly generate editor sequences that may possess editing efficiency, which are then screened and validated through experiments [37,69]. This integrated model combining “AI + the three major strategies” with “AI de novo design” will accelerate the iterative upgrading of gene-editing tools, providing stronger technological support for fields such as precision medicine, agricultural innovation, and biomanufacturing.

### 5.1 AI-driven Cas editor optimizes core toolchain

To date, artificial intelligence platforms for Cas editor optimization fall into four functionally distinct yet mutually complementary categories.The first group covers protein structure and intermolecular interaction predictors, mainly including AlphaFold2 [70], AlphaFold3 [71] and ESMFold [72]. These models resolve atomic-resolution 3D conformations of Cas monomers and Cas–sgRNA–DNA ternary complexes. They further clarify binding interactions within the PAM recognition pocket, supplying structural evidence for engineering Cas variants with high fidelity and unrestricted PAM compatibility.The second category includes sequence analyzers that quantify mutational impacts, exemplified by the ESM model suite [73] and EVOLVEpro [68,74]. Trained via self-supervised learning, these models capture amino acid co-evolution signatures. Their zero-shot virtual saturation mutagenesis quantitatively ranks variant performance. Researchers can thereby pinpoint substitutions that cut off-target risks and expand PAM coverage, greatly lowering wet-lab expenses and screening timelines.The third set comprises de novo Cas design tools built on autoregressive algorithms like ProGen [75]. Free from natural sequence templates, these models generate fully synthetic Cas scaffolds under multi-dimensional functional limits: catalytic activity, molecular mass and PAM bias [37]. They excel at designing compact mini-Cas variants to resolve AAV packaging and delivery limitations.The fourth type predicts genome editing outputs, with BE-Hive [76] and PRIDICT [77] as representative examples. Trained on large-scale editing datasets, their deep-learning architectures forecast base and prime editing efficiency, product homogeneity, and DNA repair pathway bias across diverse chromatin environments. Such predictive capacity enables fully controllable programmable editing(Table 3).In summary, these four types of AI platforms provide computational support for optimizing Cas editors across multiple dimensions, including structural analysis, prediction of mutation effects, de novo protein design, and prediction of editing outcomes. Rather than replacing wet-lab experiments, they serve as efficient pre-screening tools that narrow the experimental search space and accelerate the iterative refinement process for Cas editors.

**Table 3:** Summary of representative AI tools for gene-editing research.

Name of the Method		Model Architecture/Algorithm Used	Advantage	Limitations	Applications	Reference
**Protein Structure and Molecular Interaction Prediction Tools**	AlphaFold2	Transformer Evoformer	Accurate prediction of monomeric protein structures and efficient mining of novel editing elements (e.g., deaminases)	Limitations remain in predicting dynamic structures, complex interactions, and specific classes of molecules	Applicable to prediction of a broad range of monomeric proteins	[70]
	AlphaFold3	Pairformer Diffusion model	Model dynamic interactions of multi-molecular complexes and optimize editing systems together with in vivo delivery compatibility	Limitations remain in predicting dynamic structures, complex interactions, and specific classes of molecules	Drug design and biological research	[71]
	ESMFold	ESM-2 (MLM-based protein language model) + Folding Trunk + Invariant Point Attention (IPA) structure module	Rapid single-sequence protein structure prediction without MSA based on ESM-2, featuring fast inference and low cost, suitable for orphan proteins and artificially designed sequences, and enabling high-throughput protein structure determination	Lower prediction accuracy compared with AlphaFold series, reliance on training data, difficulty in accurate prediction of complex complexes, long sequences and protein dynamic conformations, and poor stability for predicting novel folded structures	Application in mining unknown metagenomic proteins, evaluating de novo designed proteins, high-throughput primary screening of targets and rapid annotation of mutants for lightweight high-throughput prediction in protein engineering	[72]
**Sequence Representation and Mutation Effect Quantification Tools**	ESM	MLM (Masked Language Model)	Extract deep protein features for accurate prediction of key mutation sites of editing tools	High computational resource consumption and drawbacks in long-sequence processing	Protein functional annotation and mutational effect evaluation to assist the development of novel gene-editing tools	[73]
	EVOLVEpro	PLM + Random-forest regression)	Simulate evolutionary dynamics for multi-property co-optimization of editing tools	High computational demand and limited exploration of mutational space	Optimize CRISPR nuclease activity and specificity, boost editing efficiency and reduce off-target effects via iterative evolution	[68,74]
	ProteinBERT	MLM-based protein language model	Identify protein sequence patterns and screen highly specific editing tools to reduce off-target effects	High computational cost, poor accuracy on rare proteins, and weak performance in spatial conformation-associated tasks	Protein functional annotation, prediction of interprotein binding affinity and interaction sites, and guidance for artificial protein design	[78]
	MSA Transformer	MSA-aware Transformer	Mining evolutionarily conserved sites to enhance versatility and stability of editing tools	High computational resource demand and limited adaptability	Rapid and accurate identification of similarities and differences among diverse gene sequences via multiple sequence alignment (MSA)	[79]
	ProtTrans	Transformer	Prediction of protein structure–function relationships to provide reliable basis for site-directed mutagenesis	Dependence on high-quality data and limited understanding of complex biological systems	Assistance in designing novel gene-editing tools and predicting protein function and structure	[80]
	Evo	StripedHyena	Simulation of evolutionary pathways to screen functionally advantageous variants adapted to specific scenarios	Limited applications in eukaryotic gene editing and low confidence in protein structure prediction	Generation of functional CRISPR-Cas systems and design of multi-gene transposon systems (e.g., IS200/IS605 family), with capacity to generate DNA sequences up to 1 Mb	[81]
**Novel Cas Element Generation and Design Tools**	LLM (Large Language Models)	Transformer	Optimization of sgRNA design, prediction of off-target risks, and coordination of compatibility for multi-target editing	Limited capacity for processing complex gene-editing tasks	Design of novel gene-editing systems and optimization of existing technologies	[82]
	ProtGPT2	Transformer	Generation of novel amino acid sequences to expand the diversity of artificial editing elements	Reliance solely on sequence statistical features, lack of physicochemical constraints, limited physical authenticity of generated sequences, low precision in complex function customization, and mandatory simulation and experimental validation of candidate sequences	Application in de novo protein design and sequence space mining, high-throughput generation of highly novel protein sequences, and primary screening of candidate libraries	[83]
	ProGen	Autoregressive Transformer	Directed generation of editor variants with high activity and low immunogenicity	Dependence on evolutionary homology information, poor generalization for orphan proteins, lack of atomic-level physicochemical constraints, and requirement of structure prediction and wet-lab experimental validation for output sequences	Applicability to function-driven protein design, directed generation of functional proteins such as enzymes and editor variants, and sequence expansion and property optimization of known protein families	[75]
	ProteinMPNN	MPNN (Message Passing Neural Network)	Design of amino acid sequences based on known target 3D scaffolds for Cas protein interface engineering and optimization of solubility and stability	Limited capability for function prediction and reliance on known three-dimensional protein structures	Generation of corresponding amino acid sequences based on known protein three-dimensional structures to assist engineering of proteins with specific functions	[84]
**Precision Editing Outcome Prediction Tools**	BE-Hive	Gradient-boosted regression tree + Two-layer fully-connected neural network	High-precision prediction of ABE/CBE editing efficiency and bystander editing distribution, support for single/batch target evaluation, multi-dimensional quantification of base-editing feasibility, and improvement of sgRNA screening accuracy	Dependence on large-scale base-editing experimental data, limited generalization across cell types and species, compatibility only with single-base substitution, and incapability to predict large-fragment variants and complex chromatin environments	Application in evaluation of editability for single-nucleotide variants, optimal base editor screening and rational sgRNA design to reduce trial-and-error costs of base-editing experiments	[76]
	PRIDICT/PRIDICT2.0	Attention-based bidirectional recurrent neural network (Attn-BiRNN) ensemble	Broad-spectrum prediction of diverse prime editing outcomes based on attentive bidirectional recurrent neural networks, with version 2.0 integrating chromatin epigenetic features and identifying key sequence factors via interpretable analysis to improve pegRNA prediction accuracy under complex genomic contexts	Prediction performance constrained by cell-line training data, weak generalization across environments, and insufficient accuracy for predicting ultra-long and complex genomic editing events	Application in intelligent optimization of pegRNA for prime editing, prior prediction of editing efficiency and product purity, and suitability for multi-base site evaluation and screening of clinical candidate sites	[77]

### 5.2 Multidimensional advantages of AI-powered Cas protein engineering

Leveraging the aforementioned toolchain, AI and protein language models have overcome the limitations of traditional Cas protein engineering in multiple dimensions, primarily in the following three aspects:first and foremost, AI-driven rational design effectively overcomes the coupling dilemma in traditional directed evolution—where increased editing activity is often accompanied by decreased specificity—while cutting screening cycles and experimental costs by orders of magnitude [37]. Beyond this technical advantage, PLMs enable a dual-drive strategy combining metagenomic mining and de novo design. They can cluster and annotate massive uncultured metagenomic datasets to identify novel Cas subtypes, and independently generate artificial editing scaffolds without natural evolutionary templates [85]. Most importantly, such computational pipelines support personalized therapeutic design. By integrating patient-specific genomic and epigenetic profiles, models generate editing regimens with minimal off-target risks, such as corrective edits for sickle cell anemia-causing HBB variants, accelerating clinical translation of gene therapies [86].

### 5.3 Core bottlenecks and future perspectives of AI-enabled Cas engineering

Although AI-enabled Cas engineering brings remarkable technical strengths, multiple core obstacles restrict its practical deployment and iterative optimization [87,88]. The most prominent limitation lies in uneven distribution and scarcity of standardized high-quality datasets. Global public databases lack unified formatting and quality control standards for Cas structural and functional data, hindering cross-dataset integration and transfer learning. Micro-Cas systems (Cas14, CasX) suffer from a severe shortage of resolved structures and functional profiles; meanwhile, most training data derive from in vitro cell assays instead of physiologically relevant animal and clinical samples, leading to prediction bias for in vivo applications. Another critical drawback is the black-box nature of deep protein language models, which lack explicit physical and biochemical mechanistic explanations. Though these models output high-scoring mutant candidates, they cannot clarify how substitutions alter protein conformation, DNA binding kinetics and catalytic efficiency, reducing credibility for clinical use.Furthermore, existing models only adopt single-modal training based on sequences or static structures, failing to integrate epigenetic signals including chromatin accessibility, histone marks and DNA methylation into predictive frameworks. Last but not least, wet-lab throughput becomes a bottleneck within the DBTL loop. Low automation levels for gene synthesis, transfection and sequencing delay rapid model self-correction and iteration.

Addressing the core bottlenecks of AI-assisted Cas engineering, future research will advance along two core trajectories. The primary research avenue lies in the development of multimodal gene-editing large language models represented by ChatCRISPR. Such unified intelligent systems integrate editor catalytic mechanisms, standardized sgRNA design rules, epigenomic profiles and genome-wide off-target evaluation workflows [89,90]. A complementary transformative route is the construction of unmanned self-driving autonomous labs. By seamlessly connecting PLM prediction engines with automated liquid handlers and high-throughput sequencing, the full closed-loop pipeline—algorithm design—automated testing—real-time data feedback—autonomous model updating—can be realized to fully unlock the engineering potential of CRISPR editors [91].

## 6 Synergies and future trends in gene editor protein optimization strategies

The three major conventional strategies form a progressive chain of “basic exploration -- precision optimization -- systematic integration”, which collectively establish a complete technical system for optimizing Cas genome-editing tools. As cross-layer enabling tools, artificial intelligence and protein language models run through the discovery layer, optimization layer and integration layer, and meanwhile give rise to a brand-new research paradigm of de novo AI-based protein design.

High-throughput screening and directed evolution belong to the discovery layer. This strategy does not rely on pre-solved three-dimensional protein structures. It identifies beneficial mutations and functional variants via random mutagenesis combined with large-scale screening. It provides candidate molecular libraries and site clues for subsequent engineering, and addresses the question of “which sites hold engineering potential”. At this layer, artificial intelligence enables intelligent pre-design of mutant libraries, reduces library size, eliminates numerous non-functional mutations, and improves the screening efficiency of directed evolution.

Structure-based rational design falls into the optimization layer. It takes candidate mutation information derived from the discovery layer as input. Relying on protein structural analysis and computational simulation, it accurately pinpoints key functional sites and achieves controllable precise engineering through the workflow of “site-directed mutagenesis-functional validation”, answering the question of “how to conduct targeted modification at specific sites”. On one hand, structural analysis can be performed on beneficial mutations obtained from directed evolution to clarify molecular mechanisms, and further combinatorial mutations can be designed to stack favorable performances. On the other hand, high-specificity mutations acquired by rational design can mitigate the off-target risks of variants generated by directed evolution. AI-driven structure-prediction tools supplement numerous Cas protein 3D structures without experimental resolution. They reduce the dependence of rational design on structures solved by cryo-electron microscopy or X-ray crystallography, and help predict the synergistic effects of multi-site mutations.

Multi-strategy integrated protein engineering corresponds to the integration layer. It integrates outputs from the discovery layer and optimization layer. Through module fusion and structural rearrangement, it realizes multi-trait co-optimization and functional expansion to meet comprehensive demands from complex practical application scenarios. For example, high-activity mutations from directed evolution and high-specificity mutations from rational design can be assembled into one Cas scaffold. Further fusion with deaminase effector modules generates base editors with both high activity and high specificity. Combining rationally designed miniaturization mutations and screened functional modules, researchers can develop multifunctional editors compatible with in-vivo AAV delivery (Table 4). Artificial intelligence conducts multi-objective performance evaluation for massive mutation combinations, assists the selection of module-fusion and structural-rearrangement schemes, and shortens the iteration cycle of protein engineering.

**Table 4:** Comprehensive comparison of progressive optimization strategies for three classes of Cas proteins.

Dimensions of Comparison	High-Throughput Screening and Directed Evolution	Structure-Based Rational Design	Comprehensive Protein Engineering Modifications
**Technical Positioning**	Discovery layer: basic exploration, random trial and error	Optimization layer: precise breakthroughs, controlled transformation	Integration layer: system integration, multi-objective coordination
**Core Principles**	By simulating natural evolution, we construct libraries of random mutants using error-prone PCR and DNA recombination, and then enrich advantageous mutations through high-throughput screening and multiple rounds of iteration	Based on high-resolution 3D protein structures, elucidate the mechanisms by which functional domains interact with target molecules, predict key sites through molecular simulations and validate them via site-directed mutagenesis	By combining the strengths of the first two strategies and employing techniques such as module integration, domain deletion, and sequence rearrangement, we achieve multidimensional, systematic optimization
**Core Strengths**	Independence from protein structural information, suitability for proteins with unknown structures, identification of unexpected synergistic mutation combinations, simultaneous screening of multiple metrics (activity + specificity), and support for breakthrough extensions such as PAM compatibility	Highly targeted, directly targeting the modification site to avoid wasting resources; highly interpretable, clarifying the mechanism of mutation action; highly efficient optimization, specifically addressing specific problems; capable of functional conversion (e.g., dCas9), offering broad applicability	Overcoming limitations of single-strategy approaches for multi-property synergistic improvement, establishment of an integrated workflow of "screening for leads, structure-based target identification, and engineering for implementation", multi-dimensional optimization (activity, specificity, size and function), and elimination of trial-and-error cycles and structural dependency
**Major Shortcomings**	Extensive experimental resource requirements, multi-month to multi-year experimental cycles, random screening outputs, difficulty in simultaneous optimization of multiple mutually constrained traits, lack of in-depth analysis for mutation mechanisms, and a "black box" nature	Heavy reliance on high-resolution protein–substrate complex structures, unresolved structures for most Cas homologs, inability to accurately predict multi-site synergistic effects, requirement for specialized technical platforms, and a high barrier to entry	High technical complexity requiring integration of multiple technical systems, stringent requirements for overall research and development team capacity, potential prolongation of optimization cycles, and compatibility risks during module integration
**Applications**	Preliminary optimization of novel Cas proteins with unknown structures, breakthrough expansion of the PAM recognition range, resolution of the activity-specificity trade-off, discovery of new functional sites and mutation combinations, use as a preliminary screening step in rational design	Improved specificity (off-target risk management), enhanced editing efficiency (precise regulation of functional domains), functional conversion (nucleases → transcriptional regulation), miniaturized design (adapted for AAV delivery), species-specific optimization, precise modification of proteins with known structures	Development of clinical-grade editing tools (high activity + low off-target effects), development of multifunctional editing tools (base editing/guide editing), development of miniaturized multifunctional editors, meeting comprehensive requirements for complex application scenarios, system optimization for industrialization
**Representative Achievements**	xCas9-3.7, SpG, SpRY, HiFiCas9,FrCas9(PACE/CRISPR-MACE sequential evolution)	SuperFi-Cas9, dCas9, MISER Cas9, engineered Cas12i.3, LbCas12a-RRV	hfCas12Max, Flex-Cas12a(Lbm6-RVQ)

Beyond empowering the above three conventional engineering strategies, artificial intelligence has developed an independent research paradigm of de novo protein design. Without natural Cas protein sequence templates, it directly generates completely artificial Cas scaffolds and opens new avenues for creating miniaturized editing tools with novel functions. Nevertheless, AI-generated candidate variants still require validation by conventional experimental approaches, including directed-evolution screening, structural characterization and wet-lab assays. Therefore, artificial intelligence does not replace traditional protein engineering. Instead, it builds a mutually supportive, closed-loop and iterative collaborative technical ecosystem together with directed evolution, rational design and multi-strategy integrated protein engineering.

## 7 Applications and prospects

Gene editors that have been engineered for precision, efficiency, and multifunctionality have overcome the inherent limitations of traditional technologies—such as insufficient targeting accuracy, low editing efficiency, limited functionality, and difficult delivery—significantly enhancing the stability and applicability of genetic manipulation. They offer outstanding application value and industrialization potential in fields such as precision treatment of genetic diseases, targeted crop breeding, and highly sensitive nucleic acid detection. Compared to traditional large Cas proteins, small Cas proteins such as Cas12f and CasMINI have lower molecular weights and simpler structures. They can be fully packaged into a single AAV vector for efficient in vivo delivery, effectively addressing the efficiency losses and off-target risks associated with traditional dual-vector delivery. At the same time, these small proteins combine the advantages of high editing efficiency, low immunogenicity, and a broad targeting range. Engineered small Cas proteins can achieve editing efficiencies of up to 90% in human cells without significant off-target effects or tissue toxicity, providing high-quality clinical solutions for in vivo gene therapy of intractable diseases such as rare genetic disorders and hereditary cardiovascular diseases, with broad prospects for translation. In the future, driven by interdisciplinary advancements in structural biology, AI-powered protein design, and novel biomaterials, gene editing technologies will continue to evolve and upgrade, fully unlocking the clinical potential of small Cas proteins. This will accelerate the transition of gene editing from laboratory-based technical validation to standardized, large-scale, and safe industrial implementation, thereby empowering precision medicine and driving the quality and technological advancement of the modern agricultural sector.

Despite significant progress in optimizing the CRISPR/Cas system, existing CRISPR/Cas systems remain constrained by underlying issues such as the intrinsic molecular properties of proteins, genomic sequence limitations, barriers to cellular delivery, and lagging ethical and regulatory frameworks. This results in multiple bottlenecks for translational applications: first, the functional domains of Cas proteins inherently involve trade-offs in performance, making it difficult to simultaneously achieve miniaturization, high activity, and high specificity; second, the natural PAM recognition domain limits the scope of genomic targeting, making precise editing at many sites unfeasible; third, the mismatch tolerance of sgRNAs readily induces off-target damage, making it difficult to meet clinical safety standards; fourth, in vivo delivery vectors suffer from insufficient transfection efficiency and high immunogenicity, hindering clinical translation; fifth, germline editing and gene-edited crops pose irreversible genetic and ecological risks, and the supporting ethical and regulatory frameworks remain incomplete. To address these root causes, future research should focus on five key directions: using computer-aided directed evolution to modify protein conformations, balancing molecular weight, editing efficiency, and targeting specificity; reshaping the PAM-binding pocket or developing TnpB/IscB mini-editors to eliminate target sequence restrictions; optimizing nucleic acid interaction interfaces to narrow the mismatch tolerance range and reduce off-target risks; developing low-toxicity, tissue-targeted delivery vectors to enhance in vivo administration safety; and establishing a tiered ethical review and risk management system to balance technological innovation with ethical standards.

With the advancement of cryo-EM structural analysis, the maturation of AI-based protein prediction models, and the discovery of novel natural micro-editing tools, the next generation of highly efficient, precise, and broad-spectrum gene editing systems will continue to evolve. These systems will provide core technological support for basic life science research, the clinical development of gene therapies, precision agricultural breeding, and highly sensitive nucleic acid detection, driving breakthrough innovations in these fields.

## 8 Conclusion

Improvements in the performance of CRISPR-Cas gene editing technology are highly dependent on the protein engineering of Cas proteins. Directed evolution, structure-based rational design, and comprehensive protein engineering collectively form a three-tiered, progressive technical framework for Cas protein modification: “random exploration—precise modification—system integration.” Directed evolution relies on random mutations combined with high-throughput screening to identify functional variants; it is suitable for overcoming PAM recognition limitations and resolving the performance trade-offs between activity and specificity, but suffers from drawbacks such as lengthy experimental cycles and insufficient elucidation of the molecular mechanisms underlying mutations; structure-based rational design utilizes high-resolution three-dimensional protein structures to enable site-specific modifications, allowing for targeted modifications such as off-target risk management, functional reengineering, and protein miniaturization; however, its development is constrained by bottlenecks in the structural characterization of protein-substrate complexes; integrated protein engineering, which combines the strengths of the former two approaches, establishes a closed-loop process of “initial screening to identify candidate variants—structural analysis to lock in targets—engineered integration and modification.” This approach enables the synergistic optimization of multiple traits—including activity, specificity, protein molecular weight, and in vivo delivery compatibility—and represents the mainstream technical pathway for developing clinical-grade gene-editing tools.

Artificial intelligence technologies—such as protein language models and structure prediction models—are not intended to replace traditional wet lab experiments, but rather serve as cross-layer enabling tools that span the entire Cas protein modification process. AI can not only reduce the size of mutation libraries, predict the functional effects of mutations, and evaluate gene-editing outcomes, but also enable de novo design of Cas editors, expand the sequence space of artificially engineered components, and provide new approaches to addressing the multi-objective performance trade-offs that are difficult to resolve through traditional protein engineering. It is worth noting that AI-generated candidate variants still require validation through traditional experiments such as directed evolution and structural analysis. At this stage, AI-assisted Cas protein engineering still faces practical obstacles, including a scarcity of high-quality functional datasets, insufficient model interpretability, and difficulties in incorporating epigenetic features into predictive frameworks. Overcoming these challenges will depend on the further development of multimodal gene-editing large language models and automated closed-loop experimental systems.

Existing Cas gene-editing tools still face challenges in translational applications, including protein-intrinsic trade-offs, PAM sequence constraints, the risk of off-target damage, insufficient in vivo delivery efficiency, and the need to improve ethical and regulatory frameworks. In the future, we should promote the deep integration of traditional protein engineering with artificial intelligence: on the one hand, by continuing to explore and optimize mini-Cas editors such as Cas12f and TnpB/IscB, and developing a new generation of gene-editing tools suitable for in vivo AAV delivery that balance high catalytic activity with ultra-high fidelity; on the other hand, by improving the construction of standardized Cas functional datasets, promoting the iterative integration of intelligent prediction modules with automated experimental platforms, and simultaneously establishing a tiered ethical risk management system. By pursuing a dual-track approach of technological iteration and institutional regulation, we can advance gene editing technology from basic laboratory research to industrial applications such as the clinical treatment of genetic diseases, molecular breeding in agriculture, and highly sensitive nucleic acid detection, thereby fully unleashing the application potential of gene editing technology in the fields of biomedicine and modern agriculture.

## Authors’ contributions

Project administration, W.H.; Supervision, W.H., N.L.,and W.Z.,; Conceptualization,W.H., N.L.,and W.Z., Writing—original draft preparation, X.C, W.H., W.R., and Z.J.; Writing—review and editing, X.C., W.H., N.L., W.Z., W.R., Y.M., L.J. and L.S. ; Visualization, X.C, W.R. and W.H.; Funding acquisition, W.H., N.L.,and W.Z.; Validation, L.S., Y.M., and L.J.; All authors have read and agreed to the published version of the manuscript.

## Declaration of competing interest

The authors declare that they have no known competing financial interests or personal relationships that could have appeared to influence the work reported in this paper.
